# Obituary: we mourn the passing of Professor Li Shichuo, Editor-in-Chief of *Acta Epileptologica*

**DOI:** 10.1186/s42494-026-00264-4

**Published:** 2026-07-22

**Authors:** Weijia jiang, Ying Tang, Dong Zhou

**Affiliations:** 1 West China Medical Publishers, West China Hospital of Sichuan University, 14# Third Section, Renmin Nan Road, Chengdu, 610041 China; 2 Neurology Department, West China Hospital of Sichuan University, 14# Third Section, Renmin Nan Road, Chengdu, 610041 China

Li Shichuo is a pioneer of epilepsy prevention and treatment in China, a founding father of neuroepidemiological research, and a standard-bearer for national epilepsy governance worldwide, President of the 1st and 2nd councils of China Association Against Epilepsy (CAAE), and Editor-in-Chief of *Acta Epileptologica* (2019–2026), passed away on March 7, 2026, at the age of 84 after a prolonged illness. We mourn deeply and cherish the memory of Professor.

Professor Li received numerous prestigious honors: in 2019, he was awarded Top Ten Medical Experts with Outstanding Contributions: Power of Promoting Industry Development by the Organizing Committee of Medical Professionals Summit and Physicians Daily; in 2021, he was granted the Lifetime Achievement Award by the International League Against Epilepsy (ILAE) and International Bureau for Epilepsy (IBE). He authored more than 100 high-quality papers in Chinese or English.

Professor Li’s academic contributions fall into four major aspects:

Firstly, he advanced the integration of epilepsy prevention and control into the global public health agenda. In 1997, representing the WHO, he jointly launched the Global Campaign Against Epilepsy (GCAE) with the ILAE and IBE, bringing epilepsy into the global public health spotlight for the first time [1–3]. In 1998, he proposed an initiative to reduce the global burden of epilepsy, aiming to prevent epilepsy onset, narrow the treatment gap, and improve access to medical resources. He led the first comprehensive global epilepsy governance project: Global Campaign Against Epilepsy: Update on the China Demonstration Project and CREST Study [1, 4]. In 2005, he initiated the establishment of the CAAE [5]. In the same year, he represented China to join the ILAE at the 26th IEC Congress in Paris [6]. He published the world’s first national report on the GCAE, and was the first to declare at the WHO that epilepsy is a public health problem [1]. He advocated leveraging China’s approaches and practical experience to provide reference models and contribute to the world, and was among the first to put forward the concept of national epilepsy governance [7]. In 2015, he promoted the adoption of WHA Resolution 68.20 at the World Health Assembly, the world’s first resolution specifically targeting epilepsy [8]. In 2019, he authored the WHO’s first single-disease report on epilepsy, highlighting epilepsy as an urgent public health priority [9]. Internationally, he took the lead in proposing a national comprehensive epilepsy governance framework, formulated the overall framework for national epilepsy management, established an epilepsy center model and standardized workflow tailored to China’s national conditions, and carried out continuous public awareness and education centered on International Epilepsy Care Day (June 28) [10].

Secondly, he pioneered epidemiological research on major neurological diseases in China. In 1983, in collaboration with Bruce S. Schoenberg from the US NIH, he systematically introduced modern neuroepidemiological methodologies to China, established standardized protocols for data collection and quality control, and cultivated a cohort of neurologists with professional epidemiological expertise [11]. In 1985, he published China’s first epidemiological report on epilepsy, and has continued monitoring the prevalence of epilepsy in China for four decades [12]. He conducted surveys across regions with varying levels of healthcare resources in China and was first to identified the inflection point of epilepsy prevalence nationwide. He laid the foundation for China’s first large-scale epidemiological survey on neurological diseases. Based on community cohorts in six cities, his team has consistently reported the prevalence distribution and risk factors of dozens of major neurological disorders [13]. He also led clinical trials to verify the efficacy of blood pressure control in the prevention and treatment of cerebrovascular diseases, with follow-up spanning a decade from 1987 to 1997 [14].

Thirdly, he established the foundational framework for epilepsy prevention and control in resource-limited areas of China [10]. He introduced the WHO rural demonstration project to China for the first time, confirmed the long-term efficacy of phenobarbital in resource-poor settings, and validated the efficacy and safety of valproate sodium as an alternative medication regimen [3]. He also identified preventable and modifiable risk factors for death among rural patients with epilepsy in China [15].

Finally, it had long been Professor Li’s cherished wish to establish an internationally influential English academic journal in epilepsy originating from China. Thanks to his initiative and the joint efforts of the editorial team, *Acta Epileptologica* came into being. During his tenure as Editor-in-Chief (2019–2026), the journal achieved a leap forward in academic influence and internationalization, and was successfully indexed by top international and domestic databases including ESCI, PubMed Central, Scopus, and the Core Library of the Chinese Science Citation Database(CSCD).

Having devoted his lifelong efforts to laying a solid foundation, he lit a beacon to guide future generations. The editorial office will carry forward his legacy, strive for higher standards, press ahead with full efforts to advance the journal into higher-level databases, continuously expand its academic influence, and contribute wisdom and strength to building a global community for epilepsy prevention and treatment!
